# Melatonin and Abiotic Stress Tolerance in Crop Plants

**DOI:** 10.3390/ijms24087447

**Published:** 2023-04-18

**Authors:** Roshira Colombage, Mohan B. Singh, Prem L. Bhalla

**Affiliations:** Plant Molecular Biology and Biotechnology Laboratory, Faculty of Science, The University of Melbourne, Parkville, Melbourne, VIC 3010, Australia; rcolombage@student.unimelb.edu.au (R.C.); mohan@unimelb.edu.au (M.B.S.)

**Keywords:** abiotic stress, crop plants, melatonin, ER-stress, phytomelatonin

## Abstract

Increasing food demand by the growing human population and declining crop productivity due to climate change affect global food security. To meet the challenges, developing improved crops that can tolerate abiotic stresses is a priority. Melatonin in plants, also known as phytomelatonin, is an active component of the various cellular mechanisms that alleviates oxidative damage in plants, hence supporting the plant to survive abiotic stress conditions. Exogenous melatonin strengthens this defence mechanism by enhancing the detoxification of reactive by-products, promoting physiological activities, and upregulating stress-responsive genes to alleviate damage during abiotic stress. In addition to its well-known antioxidant activity, melatonin protects against abiotic stress by regulating plant hormones, activating ER stress-responsive genes, and increasing protein homoeostasis, heat shock transcription factors and heat shock proteins. Under abiotic stress, melatonin enhances the unfolded protein response, endoplasmic reticulum-associated protein degradation, and autophagy, which ultimately protect cells from programmed cell death and promotes cell repair resulting in increased plant survival.

## 1. Introduction

Climate change is adversely affecting global agricultural productivity. Rapid and/or frequent changes in temperature and rainfall patterns result in heat waves, cold waves, floods, and drought conditions. Based on the 2007 FAO report, single or multiple stress conditions are encountered on 96.5% of the global cultivated lands [1]. Further, the predictions that global climate change will accelerate sharply over the next decade will result in a severe increase in abiotic stress-induced crop yield reduction in the near future [2].

Developing strategies to reduce crop damage due to abiotic stresses caused by climate change is imperative [3]. Developing genetically modified stress-tolerant plants, using microorganisms, and the chemical pre-treatments of seeds are some approaches being developed to enhance crop plants’ tolerance to abiotic stress.

Abiotic stress is associated with the induction of oxidative stress. Extreme and/or frequent abiotic stress conditions disturb cellular redox homeostasis, creating high oxidative stress levels, which can cause irreversible damage to the plant [4,5]. Reactive oxygen species (ROS), such as hydrogen peroxide, superoxide anions, hydroxyl radicals, and singlet and triplet oxygen, are by-products of plant cellular metabolism. As the excessive accumulation of ROS is toxic to the cell, there are in-built enzymatic and non-enzymatic scavenging mechanisms for ROS detoxification in plants. The antioxidant enzyme system includes enzymes such as superoxide dismutase, guaiacol peroxidase, catalase, ascorbate peroxidase, thioredoxins, peroxiredoxins, glutathione peroxidase, and glutathione reductase. Non-enzymatic defensive mechanisms utilise ascorbic acid, glutathione, vitamin E, and flavonoids [6]. When the rate of ROS generation is higher than detoxification, its accumulation in the cell leads to oxidative stress. Exposure to abiotic stress conditions leads to the elevation of reactive oxygen species production. ROS participate in signal transduction in minute quantities, help the plant adjust its metabolism for proper stress response, and support seed germination, photosynthesis, flowering, and delay senescence [7,8]. However, abiotic stress conditions disturb the balance between ROS production and detoxification. Cellular ROS accumulation beyond a threshold level triggers oxidative stress resulting in membrane lipid peroxidation and generating malondialdehyde, electrolyte leakage, and, ultimately, programmed cell death [9,10]. Oxidative DNA damage is another consequence of cellular ROS accumulation. This damage is caused by DNA base modification and strand breaks which change the coding ability and cellular processes [11]. Oxidative stress-induced damage and the destruction of cellular components and genetic material lead to retarded plant growth, development, reproduction, and yield loss.

Accumulated evidence reveals that oxidative stress is one of the primary causes of abiotic stress-induced damage in plant growth, development, and reproduction [5,7,12,13,14,15]. Sessile plants cannot avoid environmental stress; thus, their defensive mechanisms depend on adjusting their metabolism to survive adverse conditions. These protective mechanisms include the production of the chemical melatonin, which supports the cell in neutralizing ROS by acting as a free oxygen radical scavenger and enhancing the other existing enzymatic and non-enzymatic antioxidant activities, increasing the abiotic stress tolerance [16]. Melatonin, a natural product in plants and animals, has emerged as a novel plant growth regulator. Applying melatonin in crop plants can potentially increase food production. Its effect may be related to increased antioxidant activity, photosynthetic efficiency, and adaptability to environmental stress conditions. A recent study showed melatonin application increases the crop yield by 20% under environmental stress conditions and 18% under drought stress conditions alone. Furthermore, the photosynthetic rate of melatonin-treated plants shows an increase of 44% under drought, 42% under salt, and 48% under cold stress conditions, which ultimately translates into yield [17].

Seed priming with melatonin has been used as an effective method to increase the yield of corn, mung bean, and cucumber under organic farming without fertilizer [18]. In rice, melatonin application could enhance the genes related to sucrose transporter and nitrogen uptake under a 15% reduction in nitrogen application [19]. Accordingly, exogenous melatonin application is a promising approach to minimising the use of fertilisers in conventional and organic farming. Soybean is a major oil crop with high economic value. Abiotic stress during the seedling and seed-filling stage reduces the sustainability of soybean production. Melatonin application successfully obtains higher soybean yield with improved fatty acid contents under drought and salt stress conditions [20,21]. Similarly, in maize, seed priming with melatonin results in greater yield and better salt tolerance [22,23].

This review discusses the involvement of melatonin and phytomelatonin in stress tolerance and successful approaches in using exogenous melatonin to minimise abiotic stress-induced damage in crop plants at different stages of plant development. Furthermore, the possible involvement of melatonin in the ER stress response and a potential mechanism that could explain the increased protein homeostasis promoted by melatonin under abiotic stress conditions are also discussed.

## 2. What Is Melatonin?

**Figure 1:**
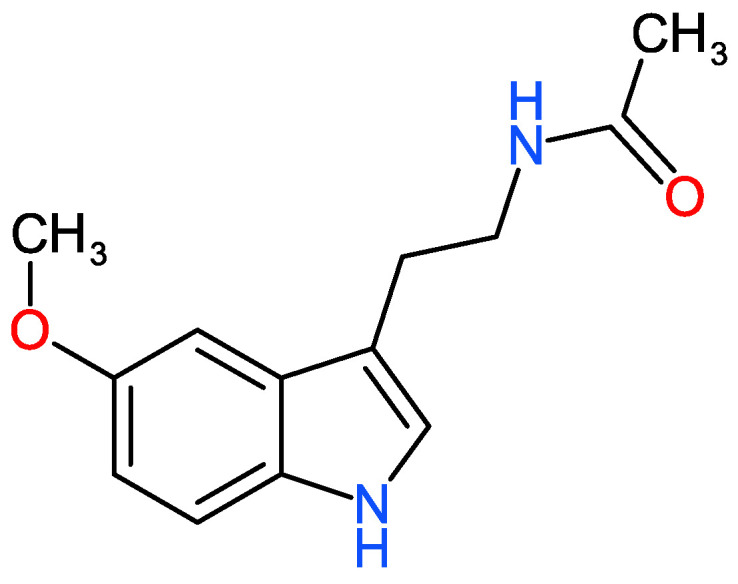
Chemical structure of Melatonin.

In the early 1900s, cow pineal gland extract that could lighten tadpole skin was discovered. Forty years later, the active compound which causes this skin-lightening effect was isolated and the compound was named melatonin because it can aggregate the melanin pigments in frogs’ melanocytes [24]. N-acetyl-5-methoxytryptamine, commonly known as melatonin, is an indolamine with the chemical formula of C_13_H_16_N_2_O_2_ (Figure 1).

Melatonin was considered present only in animals and humans for a long time. Melatonin was first discovered in plants in 1995 (Dubbels et al., 1995), reporting the presence of melatonin in nine plants in different concentrations [25]. The fact that potato does not contain melatonin suggests that exceptions are possible [26]. Considering the importance and endogenous presence of melatonin in plants, the term “Phytomelatonin” was coined in 2004 [27,28].

Melatonin in photosynthetic prokaryotes suggests that melatonin has been present in living organisms since the beginning of life [29]. According to endosymbiotic theory, eukaryotic plant cells evolved from an archaeal cell engulfing photosynthetic cyanobacteria; which later became chloroplast, and proteobacteria capable of aerobic respiration; which later became mitochondria [30]. Because the location of melatonin biosynthesis in plants is chloroplasts and mitochondria, it has been proposed that plants gained their ability to synthesise melatonin from endosymbionts [31].

### 2.1. Melatonin Biosynthesis in Plants

In animals, melatonin is synthesised in the pineal gland. However, in plants, there is no specific location for melatonin synthesis; therefore, varying levels of melatonin have been detected in different organs of plants [32]. While flowers are reported to have the highest amount of melatonin, fruits and roots have the lowest levels [33]. Even though the melatonin synthesis pathway in plants is slightly different from animals, both plants and animals use tryptophan as the precursor for melatonin biosynthesis. For melatonin synthesis, While plants use tryptophan synthesised through the shikimic acid pathway as a precursor, animals obtain tryptophan through dietary sources [34].

**Figure 2:**
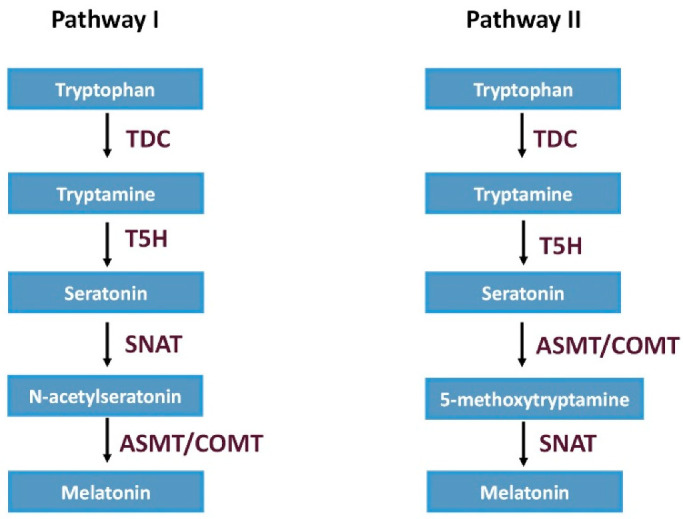
Melatonin biosynthesis pathways in plants.

There are two known pathways in plant melatonin synthesis (Figure 2). Pathway I (Tryptophan→Tryptamine→Seratonin→N-acetylseratonin→Melatonin) is most common under usual conditions, while Pathway II (Tryptophan→Tryptamine→Seratonin→5-methylhydroxytryptamine→Melatonin) is believed to become prominent during stress conditions [33,35]. There are six enzymes involved in melatonin biosynthesis in plants: Tryptophan decarboxylase (TDC), Tryptophan Dihydroxylase (TPH), Tryptamine 5-hydroxylase (T5H), Serotonin N-acetyltransferase (SNAT), acetylserotonin O-methyltransferase (ASMT), and Caffeic acid 3-O-methyltransferase (COMT) [36].

The enzymes for the respective steps are TDC, tryptophan decarboxylase; TPH, Tryptophan Dihydroxylase; T5H, tryptamine 5-hydroxylase; SNAT, serotonin N-acetyltransferase; ASMT, acetylserotonin O-methyltransferase; COMT, caffeic acid 3-O-methyltransferase. Pathway I shows greater activity under optimum conditions while Pathway II becomes prominent under stress.

### 2.2. Functions of Melatonin

Melatonin is a scavenger for reactive oxygen species (ROS) produced during metabolism in bacteria. In plants, the principal function of melatonin as a ROS scavenger remains conserved with added roles in plant growth, circadian rhythm, and stress response [29]. Considering the chemical similarity in structure and common precursor with indole acetic acid and functions in seed germination, root and shoot growth, fruiting, and senescence, it was predicted that melatonin could also function as a phytohormone [37]. The theory was confirmed by identifying the first melatonin receptor CAND2/PTMR1, which regulates the stomatal closure in Arabidopsis [38]. Recent studies have revealed in addition to its general functions in redox homeostasis, plant growth, and development, melatonin also regulates plant hormone levels and the expression of stress-responsive genes under unfavourable conditions. [39,40]. However, the exact mechanism of this multifunctional molecule in plant abiotic stress response remains to be discovered.

### 2.3. Endogenous Melatonin in Abiotic Stress

When the same species of plants grown in different environmental conditions exhibited a difference in their melatonin content, it was realised that melatonin has a role in responding to environmental changes. Further research showed that plants increase their endogenous melatonin levels in response to abiotic stress [41]. These elevated melatonin levels likely help the plant cope with external stress and adjust its metabolism to survive through it.

**Figure 3:**
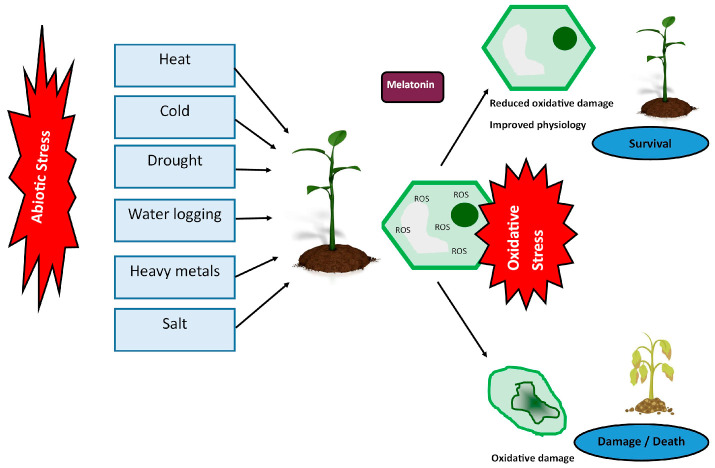
Role of melatonin in alleviating abiotic stress-induced oxidative damage in plants.

Melatonin directly improves plant tolerance towards oxidative stress by scavenging ROS, inducing existing enzymatic and non-enzymatic antioxidant activities [16,42]; indirectly, by promoting physiological functions, root development, shoot development, and activating seed germination in association with plant growth regulators [39] and photosynthesis [43]. Melatonin also decreases membrane lipid peroxidation during oxidative stress, resulting in low malondialdehyde levels and electronic leakage in plant cells, protecting the cell from programmed cell death [44]. The combined effect of all the protective metabolic alterations mediated by elevated melatonin levels reduces the abiotic stress-induced damage to the plant (Figure 3).

Abiotic stress enhances the accumulation of ROS, which creates oxidative stress. Oxidative stress damages plant cells by membrane lipid peroxidation and cell death, resulting in retarded growth, reduced yield, and/or plant death. Melatonin alleviates oxidative stress in plants and improves physiology resulting in enhanced abiotic stress tolerance.

To confirm that phytomelatonin mediated enhancement of stress tolerance in plants, scientists exposed transgenic plants that either overexpress or silence gene encoding enzymes involved in the final step of phytomelatonin biosynthesis to different stresses. These studies revealed that overexpressing phytomelatonin biosynthesis genes enhances abiotic stress tolerance. Melatonin-deficient plants created through silencing phytomelatonin biosynthesis genes show enhanced susceptibility toward abiotic stress (Table 1).

## 3. Exogenous Melatonin Application Enhances Abiotic Stress Tolerance

With the drastic changes in climate conditions, endogenous mechanisms operating in plants for stress tolerance are not strong enough to protect crop productivity. Based on the knowledge of the activity of phytomelatonin against abiotic stress, scientists have focused on applying exogenous melatonin before stress to enhance abiotic stress tolerance in plants. Exogenous melatonin pre-treatment has been demonstrated to reduce the damage due to cold, heat, heavy metals, drought, water logging, and salt stresses in plants (Table 2). The external application has been performed by soaking seed priming and adding to an irrigation/growth medium or foliar spray during the most sensitive stages in plant growth and development. In a meta-analysis conducted by Muhammad et al. (2022) using 32 studies across the globe, the exogenous application of melatonin increased the activity of SOD, POD, CAT, and APX significantly. Melatonin-induced improvement in antioxidant activity is a major underlying reason for improved plant physiology and enhanced survival under abiotic stress [56]. In most cases, 100 µM melatonin is used for foliar applications [57,58,59]. Melatonin pre-treatment in alfalfa against water logging has an optimal concentration of 100 µM, where 500 µM shows a slight decline in germination rate, CAT, and T-SOD contents [60]. However, some studies show that concentrations above optimal could be toxic to the plant [61]. In *Brassica juncea*, 0.1 µM melatonin stimulates root growth, while 100 µM shows an inhibitory effect [62]. Similarly, in red cabbage, 1 µM and 10 µM melatonin treatment show increased seed germination under copper stress, while 100 µM shows a toxic effect [63].

### 3.1. Melatonin Pre-Treatment for High-Temperature Stress

Temperature determines plant growth. Temperatures below the optimum range cause cold stress, while higher temperatures cause heat stress [89,90] Melatonin has been demonstrated to lower the adverse effect on plants during cold and heat stress conditions. Pepper (*Capsicum annum*) is an important crop plant with 25–30 °C as the optimal temperature, and temperatures below 15 °C reduce seed development and fruit set. Applying 5 µM melatonin through roots results in increased seed biomass with increased photosynthetic and antioxidant activities while reducing membrane permeability, malondialdehyde (MDA), hydrogen peroxide, and membrane permeability [64]. Melatonin pre-treatment doubled the early yield in pepper plants under chilling stress. In addition, melatonin foliar spray reduces oxidative damage and increases the net photosynthesis in two-year-old tea (*Camellia sinensis*) plants under chilling stress [65]. The optimum melatonin concentration of 100 µM for foliar spray results in a 99.5% increase in net photosynthetic rate under cold stress. Further, priming hulless barley seeds with 1 µM melatonin for 12 h before sowing could alleviate the growth inhibition caused by the cold stress [66]. Similarly, melatonin pre-treatment increased the protection towards photosynthetic pigments and reduced the membrane peroxidation in hulless barley under cold stress. In addition, melatonin treatment restored the function of circadian clock genes, which were interrupted by cold stress. Thus, it can be concluded that exogenous melatonin application can reduce cold stress damage in crop plants. Heat stress occurs in plants when the environmental temperature increases more than the optimum temperature of the plant. With the optimum concentration of 100 µM, foliar spray of melatonin on three-day-old seedlings for seven days can increase wheat thermotolerance by reducing malondialdehyde and hydrogen peroxide levels and by increasing photosynthetic pigments, proline, and enzymatic and non-enzymatic antioxidant levels [58].

Furthermore, melatonin pre-treatment induces the expression of ROS-related *TaSOD*, *TaPOD*, and *TaCAT* genes and stress-responsive *TaMYB80*, *TaWRKY26*, and *TaWRKY39* genes. The cold season crop cherry radish accumulates increased biomass when melatonin is added to the nutrient solution under heat stress [83]. The study suggests that adding an appropriate melatonin concentration to the nutrient solution can alleviate the high-temperature damage to chloroplasts and thylakoids. According to Manafi et al. (2021), 100 µM melatonin foliar spray treatment reduces the heat-induced damage to the heat-sensitive strawberry cultivar, Ventana, through an increase in the antioxidative mechanisms [57]. Moreover, increased photosynthesis and increased stress-responsive gene, *FaTHsfA2a* and *HSP90*, expression was observed. Tall fescue (*Festuca arundinacea*), a cool-season turfgrass is considered one of the most important grasses in the world and shows high susceptibility toward heat during the seedling stage. According to Alam et al. (2018), adding 20 µM melatonin to the growth medium of tall fescue at the eight-day-old seedling stage led to increased heat tolerance with increased fresh weight, plant height, chlorophyll content, protein content, and antioxidant enzymes (SOD, POD, and CAT) activities with decreased electrolyte leakage, MDA, superoxide anion, and hydrogen peroxide levels. In addition, *FaAWPM*, *FaCYTC-2*, *FaHSFA3*, *FaHSP18.2*, and *FaCML38* were upregulated, and *FaF-box*, *FaHSFA6B*, and *FaCYP710A* were downregulated with melatonin treatment under heat stress [88].

Similarly, kiwifruit (*Actinidia deliciosa*) seedlings pre-treated 5 times with 200 µM melatonin every two days could alleviate the heat-induced damage to the seedlings by increasing antioxidant enzyme activity (SOD, CAT, and POD) and reduce hydrogen peroxide levels. In addition, melatonin promotes non-enzymatic antioxidation under heat stress by increasing the ascorbic acid levels and the enzyme activities related to ascorbic acid–glutathione cycles such as ascorbate peroxidase (APX), monodehydroascorbate reductase (MDHAR), dehydroascorbate reductase (DHAR), and glutathione reductase (GR) with upregulation of more than 90% glutathione transferase (GST) genes [87].

Soybean (*Glycine max*), an important oilseed crop worldwide, is highly susceptible to stress [91], including heat-induced oxidative stress during the seedling stage. Imran et al. (2021) found that the melatonin pre-treatment works the same way in soybean seedlings under heat stress to prevent heat-induced oxidative damage with increased photosynthetic pigments and gene expression involved in enzymatic and non-enzymatic antioxidation. Furthermore, the study also showed that melatonin increases phenolics, flavonoids, proline, endogenous melatonin, salicylic acid, and polyamines (spermine, spermidine, and putrescine). Melatonin pre-treatment also reduces the abscisic acid (ABA) content in soybean seedlings by downregulating ABA biosynthesis genes and upregulating ABA catabolic genes. Further, the study also found that melatonin pre-treatment upregulated *gmHSFA2* and *gmHSP90A1* under heat stress [86].

### 3.2. Melatonin Pre-Treatment Reduces Heat-Induced Damage during Reproduction

The plant reproduction stage is highly vulnerable to temperature stress. The stress conditions occurring through the flowering and seed set stage affect the fertility and quality–quantity of the yield [92]. However, limited studies have focused on applying exogenous melatonin to minimise the adverse effects of abiotic stress during plant reproductive stages. Melatonin pre-treatment can alleviate pollen abortion in tomatoes under heat stress [85]. High temperature can alter bud morphology and pollen infertility, leading to declined yield [93]. Under heat stress, reactive oxygen species accumulate in the tapetum, which leads to pre-mature degeneration of the tapetum resulting in abnormal or infertile pollen grains [93]. The treatment of tomato plants with 50 µM melatonin enhanced the heat tolerance of the anther tissues by enhancing the expression of genes encoding antioxidant enzymes [85]. Further, the heat-induced damage to the tapetal cells is alleviated, leading to reduced damage to the unicellular microspores in tomato anthers. The mature pollen grains from the untreated heat-stressed plant show 24.8% viability and a 38.7% germination rate. In contrast, melatonin-treated plants show 45.7% viability and a 50.5% germination rate after a 3-h heat stress treatment. The study concluded that the melatonin pre-treatment alleviates pollen damage by minimising ROS accumulation in anthers, and hence can be used to protect crop plants during reproduction from abiotic stress.

Similarly, the application of melatonin as a foliar spray once, one day before the heat stress, increased the heat tolerance in rice plants during the reproductive stage [84]. However, the study focused on the chlorophyll content and photosynthesis rate, which are reported to increase in the melatonin-pre-treated plants under heat stress.

### 3.3. Melatonin Pre-Treatment for Water Stress

The amount and water quality are critical factors for plant growth and development. Water shortage leads to drought stress; excess water creates water logging/flood stress, while excessive water salinity leads to salt stress. With the appropriate concentration and proper application methods, melatonin can minimise the adverse effects on plants caused by all these three types of stress. In soybean (*Glycine max*) seedlings, melatonin application through roots produced better results than the foliar spray, while 100 µM provides better protection than 50 µM against drought stress [71]. According to the study, the reason behind this protection is increased plant biomass, photosynthetic activity, enzymatic and non-enzymatic antioxidant activity, proline and sugar content, salicylic acid, and jasmonic acid contents. The reduction in ABA, ROS, electrolyte leakage, and MDA also contributed to alleviating drought-induced oxidative damage in soybean seedlings. In contrast, applying 10 µM melatonin protects soybean seedlings from flooding stress by preventing flood-induced cell death in roots and increasing seedling length [73]. Proteomic studies have revealed that the abundance of the 13-hydroxylupanine *O*-tigloyltransferase, a protein involved in alkaloid metabolism and ROS scavenging, decreases with flooding, and comparatively increases in melatonin-treated plants during flooding stress. Further, melatonin promotes soybean seedlings under flood stress by minimising protein degradation, RNA modification, and cell wall metabolism. Like soybean, alfalfa (*Medicago sativa*) seedlings show melatonin-mediated protection in drought and waterlogging stress. A foliar spray of 100 µM melatonin increased the plant carbon, nitrogen, potassium, and calcium content under drought stress. The treatment also increased the soluble sugar, proline, and enzymatic antioxidant activity. Melatonin also reduces the levels of hydrogen peroxide, MDA, superoxide anion, and electrolyte leakage in alfalfa seedlings under drought stress [69]. Under waterlogging conditions, 100 µM melatonin foliar spray reduced the damage to six weeks old alfalfa plants by enhancing the expression of polyamine synthesis genes. It decreased the expression of ethylene synthesis genes, leading to increased polyamines and decreased ethylene, resulting in increased photosynthesis, enhanced membrane stability, and reduced leaf senescence [60]. In addition to drought and waterlogging, melatonin helps alfalfa survive through salt stress. Priming alfalfa seeds with 50 µM melatonin increases the germination rate, root and shoot growth enzymatic and non-enzymatic antioxidant activity, and decreases the hydrogen peroxide content, electrolyte leakage, and MDA content [77]. In addition, seedlings germinated from melatonin-primed seeds show relatively enhanced expression in phytomelatonin biosynthesis genes, *TDC*, *SNAT*, and *ASMT*. The sodium content of melatonin-treated seedlings was low, even without salt stress. This response can be considered as melatonin priming the plant for salt stress before the occurrence of the stress condition. Metabolome analysis of melatonin-treated rice plants shows an increased abundance of endogenous melatonin and its intermediates (5-hydroxy-L-tryptophan, *N*^1^-acetyl-*N*^2^-formyl-5-methoxykynuramine) [94]. Increased photosynthesis with melatonin supports plants in surviving water stresses. Recently, Cherono et al. (2021) showed that in coffee (*Coffea arabica*) seedlings under drought, melatonin can enhance the expression of the photosynthetic gene *RBSC2*, which encodes Rubisco protein, and suppresses the expression of chlorophyll degradation gene *PAO* encoding pheophorbide—an oxygenase [70].

### 3.4. Melatonin for Heavy Metal Stress

Heavy metals are toxic contaminants in the soil that, even in small quantities, cause adverse effects on plant growth, development, and yield [95]. In a study on wheat, cadmium stress damages wheat seedling roots and increases root hydrogen peroxide levels. As an endogenous defence system, cadmium stress induces melatonin biosynthesis gene *TaASMT* and *TaTDC* expression along with *HSFA* expression. Exogenous application of melatonin enhanced root and shoot growth, increased enzymatic and non-enzymatic antioxidant activity, and enhanced the tolerance of wheat seedlings toward cadmium stress [80]. Similarly, the application of exogenous melatonin in wheat seedlings also enhanced aluminium tolerance by reducing ROS levels, lipid peroxidation, and cell wall damage in roots [81]. In tomato seedlings, melatonin pre-treatment can minimise the negative effect caused by nickel stress [59]. According to the study, melatonin enhances nutrient uptake under nickel stress, enhances osmotic adjustment, stimulates secondary plant metabolism, increases antioxidant defence, and increases membrane integrity. In addition, it increases photosynthesis efficiency and upregulates genes involved in photosynthesis.

### 3.5. Melatonin Pre-Treatment for Combined Stresses

While many studies have shown that melatonin pre-treatment can protect plants from different abiotic stresses, plants might simultaneously face more than one abiotic stress in field conditions. However, very few studies have been conducted to evaluate the effect of melatonin pre-treatment on combined abiotic stresses. The tomato plant showed better survival with a foliar spray of 100 µM melatonin before being subjected to combined heat and salt stresses [96]. According to this study, melatonin treatment can maintain high levels of stomatal conductance and carbon dioxide assimilation and regulate the transpiration rate under heat and salinity. It also increases photosynthesis and antioxidant capacity; reduces hydrogen peroxide, lipid peroxidation, and protein oxidation.

Likewise, melatonin-pre-treated pepper plants demonstrate greater tolerance to salt stress combined with iron deficiency [97]. Similarly, melatonin treatment reduces hydrogen peroxide, MDA, and electrolyte leakage and increases the antioxidant enzyme catalase (CAT) level under individual and combined salt and iron deficiency. When pepper seedlings are subjected to low light intensity combined with low temperature, it reduces photosynthesis efficiency and negatively affects the plant biomass. Melatonin pre-treatment helps to alleviate these effects by regulating CaPsb genes involved in photosynthesis and increases overall biomass and plant survival under combined stresses [98]. Crop plants could come across biotic stresses overlapping with abiotic stress. Melatonin pre-treatment also effectively protects plants against biotic stress, including viral, fungal and bacterial pathogens, insects, and nematodes. It has been demonstrated that melatonin can be used as a substitute for pesticides [99]. Moreover, combining with melatonin increases the effect of pesticides, reducing the amount of pesticide used [100]. Biotic stress resistance provided by melatonin involves defence gene activation, ROS and NO scavenging, hormonal cross-talk and cell wall thickening of plants, and pathogen weakening.

*Phytopthera infestants* is the fungal pathogen that causes potato late blight, the greatest threat to potato growth. Applying 10 µM melatonin on leaves and tuber slices could prevent the occurrence of potato late blight by disrupting the fungal structure. In addition, the synergistic effect of melatonin and fungicide combination significantly enhances the inhibitory effect of fungicide or melatonin alone. It is demonstrated that melatonin increases fungicide susceptibility and virulence and drug resistance in *P. infestants* through differential gene expression [100]. Leaf application of 100 µM melatonin in cucumber alleviates downy mildew caused by *Pseudoperonospora cubensis*, activating antioxidant genes [101]. *Arabidopsis thaliana* exhibits enhanced resistance against the bacterial pathogen *Pseudomonas syringae* with exogenous application of melatonin by ROS and NO-mediated defence signalling, cell wall strengthening, phytohormone signalling, and pathogenesis-related gene regulation [102,103,104]. Similarly, in the crop plant cucumber, applying melatonin on leaves has improved the resistance against *Pseudomonas syringae* pv. *Lachrymans* differential expression of genes involved in signal transduction pathways [105]. Moreover, ROS scavenging mechanism provided by exogenous melatonin enhances the resistance against alfalfa mosaic virus in eggplant [106]. It has also been demonstrated that melatonin has a possible safener activity towards plants with different herbicides [107].

Collectively, it is evident that melatonin pre-treatment acts on increasing endogenous melatonin levels and regulates stress-responsive and metabolic genes resulting in decreased hydrogen peroxide, superoxide, malondialdehyde, and electronic leakage in plants under abiotic stress. Moreover, it increases osmotic regulation by increasing proline and soluble protein levels, regulating non-enzymatic antioxidants such as glutathione (GSH) and ascorbic acid (AsA); and antioxidant enzyme activity such as superoxide dismutase (SOD), peroxidase (POD), and catalase (CAT). In addition, melatonin also increases plant biomass, photosynthesis, and gas exchange under abiotic stress conditions. Recent studies showed that melatonin helps with repairing oxidative DNA damage. Overexpression of *AANAT* and *HIOMT*, genes encoding melatonin biosynthetic enzymes in mammals, makes *Nicotiana sylvestris* protoplasts less susceptible to UV-B-induced DNA damage. [108]. Exogenous melatonin alleviates the root DNA damage in *Ardisia mamillata* and *Ardisia crenata* under lead stress. The study shows that DNA protection is provided by the parallel to the enhanced antioxidant activity [109]. However, a recent study showed this antioxidant activity is not the sole mechanism underlying the defensive action of melatonin. It also has a role in gene regulation of endoplasmic reticulum stress (ER stress) response and protein protection [40].

## 4. Role of Melatonin in Cellular Function

### 4.1. ER Stress Response and Protein Homeostasis

Even though the antioxidant role of melatonin in protecting plants from stress is widely discussed, not enough attention has been given to the direct involvement of melatonin in the regulation of stress-responsive genes. The endoplasmic reticulum stress response is a natural mechanism that helps plants to survive stress conditions. The endoplasmic reticulum (ER) is a subcellular organelle responsible for protein folding [110]. Proper folding of a newly synthesised protein is essential for its function. Unfolded/misfolded proteins cannot perform their designated function and are toxic to the cell. ER has a quality control system to ensure that unfolded proteins do not leave ER. Under biotic and abiotic stress conditions, protein folding demand exceeds its capacity, which leads to ER stress. Unfolded protein response (UPR) ER’s stress response protects the cell and the whole organism [110,111,112]. Upon ER stress, UPR upregulates stress-responsive genes and enables additional rounds of protein folding to repair misfolded proteins with the help of molecular chaperones. In plants, the UPR pathway has two main arms. The bZIP28/bZIP17 arm and IRE/bZIP60 arm are ER stress signalling pathways. Improperly folded proteins beyond repair are degraded through ER-associated cell degradation (ERAD). If these actions are not enough to alleviate ER stress, it leads to autophagy, a self-degradation of cellular components mediated by autophagosomes.

In some cases, cells become highly damaged due to the accumulation of misfolded proteins that any of the protective mechanisms could not repair. Such a situation leads to programmed cell death (PCD) for the integrity of the whole organism. In addition to classical UPR pathways, mitogen-activated protein kinase (MAPK) signalling pathways also have a role in ER stress response.

Melatonin increases the protein folding capacity of cells under ER stress. As detailed by Malhotra and Kaufman (2007), ER lumen is an oxidative environment where protein oxidation occurs and releases ROS as by-products. Activation of UPR upon oxidative stress suggests the close relationship between protein folding and ROS [113]. In this case, melatonin, an endogenous chemical with known antioxidation ability, can aid ER stress response and protein protection by ROS scavenging. However, melatonin’s role in ER stress response goes beyond its antioxidant actions. In 2004, Lei et al. discovered that melatonin pre-treatment weakened cold-induced apoptosis in carrot suspension cells [114]. As the levels of ROS generation remained unaffected by melatonin pre-treatment, the study suggested that the protective role of melatonin is not directly related to its ROS scavenging ability. Instead, the “polyamines”, which were elevated with melatonin pre-treatment, were suggested to be the reason behind melatonin-induced cellular protection. In plants, polyamines, putrescine, spermidine, and spermine increase with exogenous melatonin [115,116]. Spermine induces the UPR by enhancing the expression of *bZIP17*, *bZIP28*, and *bZIP60* and activating the splicing of bZIP60 mRNA in Arabidopsis, which are essential steps in UPR [117,118].

Considering the evidence that melatonin increases the spermine level and enhances ER stress response, melatonin may involve ER stress response and protein protection in plants independent of its antioxidant activity. In favour of this hypothesis, Lee and Back (2018) found a close relationship between melatonin and the IRE/bZIP60 arm of UPR in *Arabidopsis*.

According to Lee and Back (2018), melatonin confers ER stress tolerance in Arabidopsis by increasing ER chaperones for antioxidant defence. To confirm this action, they used transgenic *Arabidopsis* lines with overexpression and knockdown of the *SNAT* gene; a gene that encodes an essential enzyme in melatonin biosynthesis in plants. Under ER stress, molecular chaperones involved in protein protection, *BIP2*, *BIP3*, and *CNX1*, are highly upregulated in *SNAT* overexpressed lines and downregulated in knockdown lines. In contrast, there was no marked difference in superoxide and antioxidant levels between wild-type and *SNAT* transgenic lines under ER stress [119]. According to the study, melatonin increases the transcription of these molecular chaperones even without ER stress, indicating that melatonin-mediated UPR is a regular activity in plants. Furthermore, *Arabidopsis* mutant plants lacking *bZIP60* genes show low levels of upregulation in molecular chaperones under ER stress and melatonin treatment. In contrast, none of the molecular chaperone levels showed much difference between wild-type and *bZIP28* knockout mutants, suggesting that endogenous melatonin-mediated ER stress response is mainly related to the IRE/bZIP60 arm of UPR. Upon the activation of this arm, *bZIP60* undergoes nonconventional splicing to produce an active transcription factor, bZIP60s. This study also showed that the splicing of *bZIP60* occurs at a higher rate in the SNAT overexpressed line and a lower rate in the *SNAT* knockdown line of *Arabidopsis* compared to the wild type. This suggests the positive involvement of melatonin in activating the IRE/bZIP60 arm of UPR in plants. In addition, exogenous melatonin repairs the damage to the ER network induced by tunicamycin, an ER stress inducer. The study further showed that melatonin treatment could fully restore ER-resident immune response chaperones, BRI1 and FLS2, in *N. benthamiana* leaves damaged by tunicamycin-induced ER stress.

The involvement of melatonin in ER stress pathways is further explored by studying the expression levels of ER stress genes under tunicamycin-induced ER stress with and without melatonin [120]. Genes involved in ER stress, *bZIP17*, *bZIP28*, *bZIP60*, *IRE1A*, and *IRE1B*, were upregulated in *Arabidopsis* upon ER stress-inducing conditions. Among those genes, *bZIP17*, *bZIP28*, *IRE1A*, and *IRE1B* decreased the transcript level to control with 25 µM exogenous melatonin applied in the growth medium/roots, suggesting that melatonin treatment eliminated the ER stress from the roots. Applying melatonin to roots did not change the transcriptional levels of these genes in shoots. While *bZIP60* was downregulated with the melatonin treatment, the expression did not reach the control levels.

### 4.2. Melatonin and Autophagy

As a part of protein and cell protection, melatonin induces autophagy under stress. Autophagy is the ultimate response to save cells from PCD [121]. Arabidopsis seedlings grown under oxidative stress show higher levels of autophagosomes, autophagy-related *ATG* gene expression, and a higher survival rate with melatonin treatment [122]. Under heat stress, melatonin also upregulated autophagy-related genes, *ATG*, in tomato leaves [53] and anthers [85]. In addition to exogenous melatonin, overexpressing of the melatonin biosynthetic *SNAT* gene increases the salt tolerance in Arabidopsis seedlings, with enhanced levels of autophagy [123].

Recent studies show that melatonin induces the expression of autophagy genes, and autophagy genes are also involved in the expression of melatonin biosynthetic genes. Overexpression of autophagy-related genes (*ATG8b*, *8c*, and *8e*) facilitated the protein expression levels of TDC2, ASMT2, and ASMT3 in cassava [124]. According to the study, exogenous melatonin promotes autophagy by inducing the expression of autophagy genes, while overexpression of melatonin biosynthesis genes *MeTDC2*, *MeASMT2*, and *MeASMT3* also induce autophagy. Although the exact mechanism of melatonin-induced autophagy is yet unknown, the reversible actions in the regulation of autophagy genes and melatonin biosynthesis genes suggest a close relationship between the two, which has the goal of helping plants to survive abiotic stress. Perhaps, like HSFA1/HSP40 with *SNAT/COMT*, autophagy genes also can promote *TDC/ASMT* gene expression.

### 4.3. Interaction of Melatonin with HSFs and HSPs

Heat shock proteins (HSP) are molecular chaperones with the role of repairing and/or degrading accumulated harmful unfolded proteins to assist the organism’s survival under stress. Heat shock factors (HSF) are transcriptional regulators of HSP [15,125].

**Figure 4:**
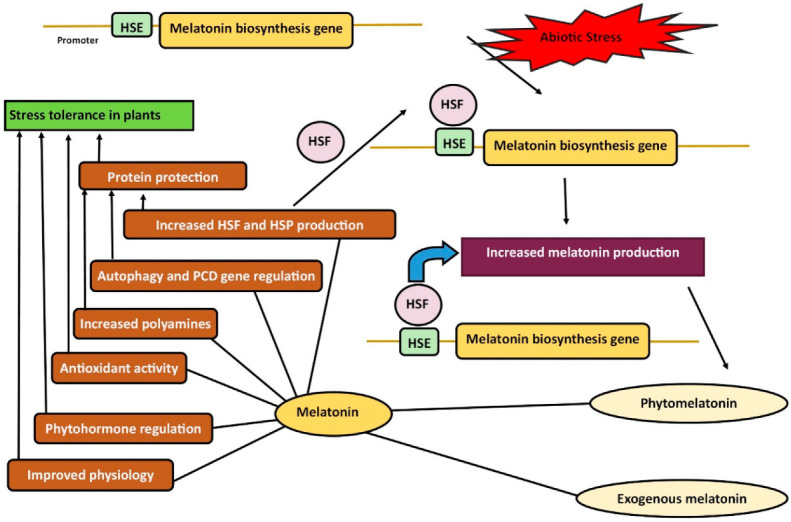
HSFs and HSPs in phytomelatonin biosynthesis under stress conditions.

It has been demonstrated that HSFs and HSPs are involved in phytomelatonin biosynthesis under stress conditions. As outlined in Figure 4, low levels of existing melatonin could be a limiting factor for plant protein protection mechanism, and hence a reason for plant abiotic stress susceptibility. Providing a boost of melatonin either by a genetic modification or external application increases protein protection and HSF levels, further upregulating melatonin biosynthesis (Figure 4).

HSF binds to the heat shock elements (HSE) in the promoter region of the melatonin biosynthesis genes to enhance the transcription. Exogenous melatonin uptake increases the total melatonin content, which stimulates melatonin biosynthesis gene expression leading to increased abiotic stress tolerance in plants.

The overexpression of *HSFA1a* in tomatoes increases the accumulation of melatonin under cadmium stress, while silencing *HSFA1a* reduces melatonin production and cadmium tolerance [126]. Overexpressing of *HSFA1a* also increases the expression of *COMT1*, a gene encoding an essential enzyme in the melatonin biosynthesis pathway, by binding to the heat shock element of its promoter under cadmium stress. Overexpressing of *HSFA1a* increases HSP production, while silencing of *HSFA1a* decreases HSP under cadmium stress showing that HSFA1a is responsible for HSP chaperone production under cadmium stress. When *HSFA1a* is overexpressed, if *COMT1* is silenced, it reduces the HSP production and cadmium tolerance along with the melatonin accumulation [126]. The study suggests that *HSFA1* upregulates *COMT1* by binding to the heat shock element in the COMT promoter to increase melatonin synthesis under cadmium stress. Increased melatonin levels further increase HSF and HSP levels for enhancing cadmium tolerance.

The interaction of *SNAT* with HSP40 promotes thermotolerance in tomatoes [48]. Downregulation of HSP40 lowers melatonin synthesis under heat stress may be due to the protection provided by HSP40 towards SNAT against heat-induced degradation. The two heat shock elements in the tomato SNAT promoter region also indicate a possibility of HSF binding the SNAT promoter and upregulating the gene expression under heat stress. Overexpressing of SNAT increased the synthesis of melatonin, HSP40, HSP20, HSP21, HSP90, and HSP17 [48]. The study suggested that HSP40 promotes melatonin synthesis/protection and enhances thermotolerance by reducing oxidative stress.

ASMT is another enzyme involved in melatonin biosynthesis. The overexpression of ASMT in tomato plants upregulates HSP17.7, HSP20-1, HSP21, HSP70, and HSP90 under heat stress resulting in increased thermotolerance [53]. The foliar application of exogenous melatonin shows the same results as the ASMT overexpression. The upregulation of HSPs results in refolding denatured proteins under heat stress with a drastic reduction in protein aggregation compared to the wild type. These results showed that melatonin enhances protein protection under heat stress, either as phytomelatonin or exogenous melatonin [53].

In Arabidopsis, under heat stress, exogenous melatonin showed increased upregulation in *HSFA1*, *HSP90*, and *HSP101* while silencing the *HSFA1* gene alleviating the melatonin-induced thermotolerance [127], suggesting that the relationship between HSFA1 and melatonin is bi-directional. Under heat stress, melatonin-pre-treated tomato anthers showed increased upregulation in the expression of HSP21 and HSP70 with reduced pollen damage [85]. The study showed that melatonin had reduced the oxidative stress in tomato anthers; the upregulation of *HSP21* and *HSP70* significantly protects anther development under heat stress. In kiwifruit, ten HSPs, including HSP70 and three small HSPs, were upregulated with melatonin pre-treatment under heat stress [128]. It is reported that when tall fescue is treated with melatonin before the heat stress, it upregulates *HSFA3* but downregulates *HSFB2B*. In addition to *HSFA3*, melatonin pre-treatment upregulates *AWPM*, reported to have a role in rice drought stress tolerance [129], CYTC-2, and CML 38, reported to have a role in plant growth and development [88]. Melatonin spray on chrysanthemum plants enhanced thermotolerance by increasing the upregulation of *HSP90*, *HSP23*, *HSP80*, *HSP70*, *HSFS-3*, *HSFA-2b*, and *HSFA1a* in addition to genes involved in the calcium signal transduction, phytohormone regulation, and carbohydrates metabolism and photosynthetic pigments. [130]. The expression of HSP90, HSFA2a, and HSFB1a was increased by melatonin pre-treatment in strawberry plants under heat stress [57].

Accordingly, an increase in melatonin levels strengthens abiotic stress tolerance by boosting HSF/HSP levels, while reduced endogenous melatonin reduces the capability to produce HSP. Genes encoding SNAT and COMT, major enzymes essential for the final step of melatonin biosynthesis, contain stress-responsive elements in the promoter region, facilitating HSF/HSP expression under abiotic stress [48,126]. The binding of HSF/HSP enhanced the melatonin biosynthesis gene regulation, leading to increased melatonin levels, promoting antioxidative abilities and protein protection, and enhancing the production of heat shock transcription factors and molecular chaperones, HSF and HSP. Exogenous melatonin application and the overexpression of melatonin biosynthesis genes promote this process leading to increased abiotic stress tolerance. This indicates that a lower level of melatonin present in plant tissues could be the limiting factor for this process of melatonin-mediated molecular chaperone production under abiotic stress. Lower HSP production in *HSF*-overexpressed, *COMT*-silenced plants [126] also suggests that even with a high level of HSF, melatonin is necessary for the sufficient production of HSP to provide protein protection.

## 5. Melatonin Regulates Phytohormones under Abiotic Stress

In regulating plant growth and development, melatonin also alters the levels of hormones during unfavourable conditions. Recent studies show that melatonin acts as a regulatory hub of phytohormones under normal and stressful conditions [39]. Interestingly, depending on the stress condition and plant growth stage, melatonin levels regulate plant hormones in contrasting ways to support the plant’s survival.

Recent studies have shown that the increased melatonin levels reduced abscisic acid (ABA) levels under drought but enhanced ABA levels under cold stress. However, in both cases, melatonin enhances stress tolerance in plants. Jahan et al. (2021) showed that in tomato seedlings, exogenous melatonin increased the gibberellic acid (GA) and decreased the abscisic acid (ABA) levels to alleviate heat-induced leaf senescence [82]. Similarly, melatonin promoted the germination of cotton seeds under drought stress by modifying the drought-induced reduction in GA and promoting ABA [72]. A study on coffee also supported the observation that melatonin reduces ABA levels during drought via downregulating the ABA-responsive binding element protein (AREB) in coffee leaves under drought stress [70].

In contrast, melatonin increases ABA levels in barley under drought-primed cold stress leading to better water status maintenance and higher survival [131]. The study used drought-primed wild-type and ABA-deficient mutant barley plants and showed that drought priming increased endogenous melatonin and ABA in the wild type but not in ABA deficient mutant. Exogenous melatonin (foliar and through roots) treatment with drought priming further increased endogenous ABA levels when the wild-type plants are subjected to cold stress. Interestingly, cold-stressed melatonin-treated ABA deficient mutant showed increased ABA levels indicating that melatonin is an important signalling molecule that regulates plant hormones in a stress-dependent manner.

Similarly, exogenous melatonin also increases the ABA levels and cold-responsive CBF genes in *Elymus nutans*, promoting cold tolerance [132]. Further, the study by Zhao et al. (2017) demonstrated that melatonin pre-treatment mitigates the chilling stress damage in cucumber seedlings by upregulating ABA biosynthesis genes and downregulating ABA catabolic genes during the first four days after treatment. In addition to ABA-related genes, melatonin regulates *CaZat12*, an important stress-responsive and polyamine metabolic gene was also reported [115].

The melatonin-induced hormone regulation adjustments do not solely depend on the stress condition. They also can act differentially during the plant growth stages of plants. Under normal conditions, melatonin pre-treatment reduces the ethylene rate in lupin seedlings and ABA, another plant hormone inhibiting normal growth and development during the vegetative stage. Nevertheless, the levels of auxins, cytokinin, and gibberellin, which stimulate vegetative plant growth, increased by melatonin in the study [133].

In contrast, melatonin treatment increased the ethylene levels in tomatoes, and increased ethylene and ABA in grape berry fruit, promoting ripening and improving the quality of the fruit under normal conditions [134,135]. However, at the postharvest stage, melatonin inhibits ethylene synthesis in fruits allowing more extended storage and delaying postharvest senescence in apples and pears—in pears, melatonin inhibits ethylene synthesis by increasing nitric oxide, while in apples, the reduction in ethylene occurs parallel to the enzymatic antioxidative activity [136,137].

Further, melatonin enhances the ethylene levels in grapevine plants under salt stress by promoting ethylene precursor 1-aminocyclopropanr-1-carboxylic acid (AAC), enhancing salt tolerance [138]. This study also showed that melatonin promoted ethylene synthesis through AAC under salt stress but suppressed *MYB108A*, an essential gene involved in the ethylene synthesis pathway reducing melatonin-induced salt tolerance. The study concluded that melatonin-induced salt tolerance in grapevine is mediated by ethylene. [138].

The overexpression of corn ASMT increased the indole acetic acid (IAA) levels, leading to improved drought tolerance via enhanced lateral root formation [55]. Recently, Yang et al. (2022) studied the transcriptome of tomatoes and found that melatonin-induced drought stress tolerance was involved in the melatonin-mediated regulation of plant hormones [139]. The study also found that drought stress highly upregulated ethylene genes in tomato plants. However, in melatonin-pre-treated plants, the expression levels of ethylene genes were either similar or less to the non-stressed control plants. Similarly, drought stress also upregulated genes encoding ABA, while melatonin-pre-treated plants show less upregulation of ABA genes when subjected to drought stress. In contrast, IAA encoding genes were downregulated in non-treated plants when subjected to drought, and melatonin pre-treatment further decreases the expression of IAA genes.

## 6. Concluding Remarks

Yield reduction due to abiotic stress in crop plants affects global food security, making it vital to focus on enhancing abiotic stress tolerance in crop plants. The increase in the level of phytomelatonin during abiotic stress supports plant survival by minimising oxidative stress and enhancing physiological activities. It is well known that exogenous melatonin application during the vulnerable stages of the plant increases abiotic stress tolerance. Applying melatonin before abiotic stress during sensitive stages is a cost-effective solution to increase plant stress tolerance and minimise yield loss due to abiotic stress. Moreover, the application of exogenous melatonin is not genotype-specific and is readily applicable for large-scale crop production.

Many recent studies have focused on protecting plants during the seedling stage. Researchers have also used melatonin to increase seed germination under abiotic stress (Table 2). However, only a few studies on using melatonin during flowering or reproduction have been conducted. Successful sexual reproduction is critical for crop yield [140]. A few reports confirmed that melatonin treatment in the flowering stage increases the crop yield under abiotic stress [64,84], but studies on the effect of exogenous melatonin on the abiotic stress-induced reproductive stage are minimal. A study has shown that pre-treatment of roots with melatonin at the flowering stage can alleviate heat-induced pollen abortion in tomatoes [85], opening opportunities for further research on the effect of exogenous melatonin application during the flowering stage for enhanced stress tolerance and increased yield.

In many cases, the melatonin pre-treatment is applied during or just before the stress treatment. The long-term effects of melatonin treatment on unpredicted stress conditions are yet to be investigated. In some cases, a foliar application was performed once, just before the stress treatment [60,84], while some studies use repetitive application [57,58,141]. It is more common to use more than one application on roots before or during stress [74,85,86]. However, seedlings germinated from melatonin-primed seeds show increased abiotic stress tolerance, suggesting that single melatonin application could have a lasting effect [66,72,77,79]. Priming seeds with melatonin has been widely used to improve seed germination under abiotic stress conditions such as temperature stress, flood, and salt stress in crops such as rice, soybean, and cucumber, which are usually grown on large-scale [71,142,143,144,145,146].

Recent studies showed that exogenous melatonin promotes phytomelatonin biosynthesis in plants by upregulating genes in the melatonin biosynthetic pathway. Improving crop plants’ growth, development, and yield could indicate that these increased phytomelatonin levels might also have a long-term effect on improving abiotic stress tolerance. Hence it is essential to investigate how melatonin pre-treatment can be used to prepare crop plants for unpredictable sudden stress conditions.

The effect of exogenous melatonin application has similar results to genetically modified plants with overexpressed melatonin biosynthetic genes under abiotic stress conditions. This includes increased survival with greater fresh weight, root length, photosynthesis, and antioxidant activity. However, compared to crops genetically modified to increase melatonin production, exogenous application of melatonin is a less time-consuming and cost-effective method to improve plant abiotic stress resistance.

The application of melatonin in effectively improving plant abiotic stress tolerance is not limited to agricultural and horticultural crops. However, studies conducted on melatonin application in forestry are minimal. Root application and needle spray of exogenous melatonin have been reported to increase the seedling growth and elemental contents in Anatolian black pine (*Pinus nigra*) [147]. Salinity and alkalinity in the soil is a major stress condition that leads to afforestation. Exogenous melatonin application increases the salt and alkaline stress tolerance in poplar plants (*Populus cathayana* × *canadansis*) under greenhouse conditions [148]. Therefore, it is essential to study further the effect of exogenous melatonin in forestry.

Various studies have concluded that enhanced abiotic stress tolerance is based on the antioxidative actions of melatonin. However, melatonin has been shown to regulate stress-responsive genes and increase protein/cell protection independent of its antioxidant activity, such as ER stress responses; UPR, ERAD, and autophagy [119,122]; preventing apoptosis/PCD [114] by bringing ER stress levels down [120]; repairing subcellular organs and genetic materials [109,119]; and promoting its recovery to enhance stress tolerance as a whole organism. Further, research is needed on the melatonin–ER stress relationship and the role of polyamines in melatonin-induced ER stress response in plants.

## Figures and Tables

**Table 1:** A summary of the transgenic approaches to modulate the phytomelatonin biosynthesis pathway in abiotic stress tolerance. COMT—caffeic acid 3-O-methyltransferase, SNAT—serotonin N-acetyltransferase, ASMT—acetylserotonin O-methyltransferase.

Gene	Transgenic Plant	Transgenic Approach	Stress	Results	Reference
*COMT*	Arabidopsis (*Arabidopsis thaliana*) and Watermelon (*Citrullus lanatus*)	Overexpression—*COMT* gene (*CICOMT* from watermelon) driven by *35S* promoter	Salt	Significant increase in melatonin content. Increased survival rate under salt, freeze and mannitol-induced drought stress.	[45]
			Cold		
			Drought		
	Tomato (*Solanum lycopersicum*)	Overexpression—*SICOMT1* driven by *35S* promoter.	Salt	Elevated melatonin levels. Reduced drooping and wilting of leaves. Reduced superoxide and hydrogen peroxide levels. Increased proline levels.	[46]
	Arabidopsis (*Arabidopsis thaliana*)	Overexpression—*COMT* (*TaCOMT* from wheat) driven by *35S* promoter	Drought	Elevated melatonin levels. Increased fresh weight, lateral root number, and total root length.	[47]
*SNAT*	Tomato (*Solanum lycopersicum*)	Overexpression—*SlSNAT* gene driven by *35S* promoter.	Heat	Elevated melatonin levels. Increased chlorophyll fluorescence (Fv/Fm). Reduced wilting after 1 day recovery.	[48]
	Arabidopsis (*Arabidopsis thaliana*)	Overexpression—*SNAT* gene (*MsSNAT* from alfalfa) driven by *35S* promoter.	Cadmium	Slightly elevated melatonin levels. Reduced root length decline and lowered cadmium deposition in roots. Reduced levels of miR398, indicating low oxidative stress	[49]
		Overexpression—*SNAT* gene (*VvSNAT1* from grapes) driven by *35S* promoter.	Salt	Significantly elevated melatonin content. Improved growth potential and greener leaves.	[50]
		Overexpression—*AtSNAT* gene And *SNAT* mutant (SALK_032239)	UVB	Overexpression: Early and increased response in positive UV-B signalling regulatory genes. SALK_032239: Slow response under high and prolonged UVB treatment.	[51]
	Cucumber (*Cucumis sativus*)	Overexpression—*CsSNAT* And Silencing—*CsSNAT* (RNAi)	Salt	Overexpression: Elevated melatonin content Less ROS accumulation and upregulation of antioxidant enzymes. Increased maximum photochemical efficiency of PSII (Fv/Fm). Silencing: Lowered melatonin content. Increased ROS accumulation and decreased antioxidant enzyme activity. Lowered maximum photochemical efficiency of PSII (Fv/Fm).	[52]
*ASMT*	Tomato (*Solanum lycopersicum*)	Overexpression—*SlASMT* gene driven by *35S*	Heat	Reduced wilting Reduced electrolyte leakage. Improved photosynthesis (Fv/Fm). Decreased insoluble and ubiquitinated proteins. Enhanced expression of heat shock protein genes and several autophagy genes. Decrease in accumulation of aggregated proteins.	[53]
	Tobacco (*Nicotiana tabacum*)	Overexpression—*ASMT* (*MzASMT1* from crab apple) driven by *35S* promoter	Salt	Elevated melatonin contents. Lowered leaf wilting. Increased fresh weight, plant height, root length and relative water content. Higher chlorophyll content and improved photosynthesis (Fv/Fm).	[54]
	Arabidopsis (*Arabidopsis thaliana*)	*MzASMT* driven by *35S* promoter has been transformed into Arabidopsis.	Drought	Elevated melatonin levels. Enhanced lateral roots. Increased fresh weight.	[55]

**Table 2:** A summary of exogenous melatonin application before abiotic stress treatment in enhancing abiotic stress tolerance.

Stress	Plant	Growth Stage	Melatonin Application		Activity under Stress (Compared to Plants without Exogenous Melatonin Stressed)	Reference
			Optimised Concentration	Application Method		
Cold	Pepper (*Capsicum annuum*)	Seedling	5 µM	Soil drench with 25 mL melatonin solution one day before chilling stress	Decreased hydrogen peroxide, malondialdehyde contents and membrane permeability. Increased photosynthesis, water relation and antioxidant enzyme activity.	[64]
		Flowering	5 µM	Soil drench with 100 mL melatonin solution	Increased the yield when applied during flowering.	[64]
	Tea (*Camellia sinensis*)	Seedling (two-year-old)	100 µM	Foliage spray—three times with five-day intervals. (last treatment—24 h before the stress)	Increased antioxidant enzyme activity and stimulate photosynthesis.	[65]
	Barley (*Hordeum vulgare*)	Seeds	1 µM	Soaked the seeds for 12 h before germination.	Decreased malondialdehyde and soluble sugar content. Promoted seedling growth, increased chlorophyll content.	[66]
Drought	Rapeseed (*Brassica napus*)	Seedling	100 µM	Irrigation with 200 mL melatonin solution per pot each day for seven days.	Decreased malondialdehyde and hydrogen peroxide. Regulated leaf stomatal activity. Increased root growth and catalase activity.	[67]
	Tomato (*Solanum lycopersicum*)	Young plants (5 weeks old)	20 ppm	Foliar application	Decreased malondialdehyde and hydrogen peroxide. Increased yield and ascorbic acid content in fruits.	[68]
	Alfalfa (*Medicago sativa*)	Seedlings	100 µM	Sprayed at dark, two days before the stress and repeated every three days up to 20 days.	Increased chlorophyll and carotenoid contents, photosynthetic rate and stomatal conductance, soluble sugar and proline content. Decreased malondialdehyde, hydrogen peroxide, electrolyte leakage and superoxide anion.	[69]
	Coffee (*Coffea arabica*)	Seedlings	100 µM	Foliar spray of 20 mL and soil application of 30 mL three times per week.	Suppressed chlorophyll degradation and increased photosynthesis. Decreased malondialdehyde and electrolyte leakage. Increased enzymatic antioxidant activity.	[70]
	Soybean (*Glycine max*)	Seedlings	100 µM	Rhizosphere application	Increased chlorophyll content, photosynthetic activity, shoot and root growth, enzymatic antioxidation. Increased salicylic and jasmonic acid content. Decreased malondialdehyde, electrolyte leakage and hydrogen peroxide.	[71]
	Cotton (*Gossypium hirsutum*)	Seeds	100 µM	Soaked the seeds for 24 h prior to germination.	Increased soluble sugar and proline content. Increased stomatal regulation, germination rate, germination potential and fresh weight. Decreased the hydrogen peroxide, superoxide anion and malondialdehyde.	[72]
Water Logging	Soybean (*Glycine max*)	Seedling	10 µM	Root application at the same time of flood.	Increased root growth and development, increased root cell wall lignification. Increased alkaloid metabolism and ROS scavenging.	[73]
	Alfalfa (*Medicago sativa*)	Seedling	100 µM	Foliar spray one day before to the stress	Increased plant growth and photosynthesis. Increased endogenous melatonin levels. Increased polyamines and decreased ethylene. Decreased membrane damage and leaf senescence.	[60]
	Peach (*Prunus persica*)	seedlings	200 µM	Applied to soil every other day during the stress.	Increased root and shoot development. Positive development in photosynthetic and stomatal apparatus. Increased antioxidant activities. Increased anaerobic respiration through enhanced aerenchyma.	[74]
Salt	Tomato (*Solanum lycopersicum*)	Seedling	1 µM	Applied to the medium mixed with saline treatment	Increased photosynthesis and antioxidant enzyme activity. Decreased malondialdehyde and hydrogen peroxide content.	[75]
	Olive (*Olea europaea*)	Seedling	100 µM	Foliar spray	Increased shoot and root growth, photosynthetic pigments, proline and soluble sugars. Increased enzymatic antioxidation. Decreased hydrogen peroxide, malondialdehyde and electrolyte leakage.	[76]
	Alfalfa (*Medicago sativa*)	Seeds	10–100 µM	Seeds immersed and air-dried prior to germination	Increased seed germination, root length, seedling growth and enzymatic anti-oxidation.	[77]
		One-month-old plants	50 µM	Foliar spray every night	Decreased hydrogen peroxide, malondialdehyde and electrolyte leakage	
	Rice (*Oryza sativa*)	Seedlings	20 µM	Applied for 24 h prior to salt stress	Increased root and shoot growth. Increased the expression of stress-responsive genes.	[78]
	Rapeseed (*Brassica napus*)	Seeds	50 µM	Seed primed for 8 h prior to germination	Decreased hydrogen peroxide and superoxide anions. Increase the regulation of antioxidant enzymes, chlorophyll content, photosynthetic rate and proline content. Improved the oil quality.	[79]
Cadmium	Wheat (*Triticum aestivum*)	Seedling	0.5–100 µM	Applied directly to the roots of seedlings growing in Petri dishes	Increased root and shoot growth, Increased enzymatic and non-enzymatic anti-oxidants. Decreased hydrogen peroxide content.	[80]
Aluminium	Wheat (*Triticum aestivum*)	Seedling	10 µM	Treated for 12 h prior to the stress	Increased enzymatic and non-enzymatic antioxidant activity	[81]
Nickel	Tomato (*Solanum lycopersicum*	Seedling	100 µM	Foliar sprayed with 80 mL solution with 3 days interval during the stress	Improved photosynthesis and gas exchange. Increased enzymatic anti-oxidation. Upregulation of stress-responsive genes. Decreased hydrogen peroxide, malondialdehyde and electrolyte leakage	[59]
Heat	Tomato (*Solanum lycopersicum*)	Seedling	100 µM	Foliar sprayed every two days for seven days one week before the stress	Increased photosynthesis and stomatal activity. Decreased hydrogen peroxide, malondialdehyde and electrolyte leakage. Downregulation of genes encoding ROS accumulation.	[82]
	Wheat (*Triticum aestivum*)	Seedling	100 µM	Sprayed 80 mL of melatonin solution on leaves each day for seven days one week before the stress.	Increased chlorophyll content, enzymatic and non-enzymatic antioxidant activity and proline content. Decreased hydrogen peroxide and malondialdehyde.	[58]
	Cherry radish (*Raphanus sativus*)	Seedling	29.0 mg/L	Applied on roots mixed with Hogland’s nutrient solution.	Increased chlorophyll, carotenoid content and enzymatic antioxidation. Decreased malondialdehyde.	[83]
	Strawberry (*Fragaria × ananassa*)	Young plants (3 weeks old)	100 µM	Foliar spray three times at one-day intervals (last treatment—10 h prior to the stress)	Increased enzymatic and non-enzymatic antioxidant activity. Upregulation of stress-responsive genes. Decreased hydrogen peroxide, and malondialdehyde.	[57]
	Rice (*Oryza sativa*)	Flowering	200 µM	Sprayed one day before the stress treatment.	Increased chlorophyll content and stomatal conductance.	[84]
	Tomato (*Solanum lycopersicum*	Flowering	20 µM	Applied on roots on each day for 7 days followed by the heat stress.	Alleviated pollen abortion. Increased stability of tapetum cells and avoid pollen deformity by inducing stress-responsive genes. Increased ROS scavenging and enzymatic antioxidant activity.	[85]
	Soybean (*Glycine max*)	Seedling	100 µM	Applied on root zone (30 mL) twice daily for 6 days prior to stress.	Increased chlorophyll content and non-enzymatic anti-oxidation. Decreased hydrogen peroxide, superoxide, malondialdehyde and electronic leakage.	[86]
	Kiwifruit (*Actinidia deliciosa*)	Seedling	200 µM	Treated five times every two days prior to stress.	Increased proline, enzymatic and non-enzymatic antioxidant activity. Reduced hydrogen peroxide.	[87]
	Tall fescue (*Festuca arundinacea* Schreb.)	Seedling	20 µM	Seedlings were transferred to MS medium containing melatonin two days prior to the stress.	Increased antioxidant enzyme activity and chlorophyll content. Changed stress-responsive gene regulation. Reduced hydrogen peroxide, superoxide anion, malondialdehyde and electronic leakage.	[88]

## Data Availability

All data generated in this study are available in the article.
